# Identification of novel hypertension biomarkers using explainable AI and metabolomics

**DOI:** 10.1007/s11306-024-02182-3

**Published:** 2024-11-03

**Authors:** Karthik Sekaran, Hatem Zayed

**Affiliations:** 1 Luxembourg Centre for Systems Biomedicine (LCSB), University of Luxembourg, Esch-sur-Alzette, Luxembourg; 2 Department of Biomedical Sciences, College of Health Sciences, QU Health, Qatar University, Doha, Qatar

**Keywords:** Biomarkers, Explainable artificial intelligence, Hypertension, Metabolomics, Qatar Precision Health Institute-Qatar Biobank, Shapley additive explanations, Vanillylmandelic acid

## Abstract

**Background:**

The global incidence of hypertension, a condition of elevated blood pressure, is rising alarmingly. According to the World Health Organization’s Qatar Hypertension Profile for 2023, around 33% of adults are affected by hypertension. This is a significant public health concern that can lead to serious health complications if left untreated. Metabolic dysfunction is a primary cause of hypertension. By studying key biomarkers, we can discover new treatments to improve the lives of those with high blood pressure.

**Aims:**

This study aims to use explainable artificial intelligence (XAI) to interpret novel metabolite biosignatures linked to hypertension in Qatari Population.

**Methods:**

The study utilized liquid chromatography-mass spectrometry (LC/MS) method to profile metabolites from biosamples of Qatari nationals diagnosed with stage 1 hypertension (n = 224) and controls (n = 554). Metabolon platform was used for the annotation of raw metabolite data generated during the process. A comprehensive series of analytical procedures, including data trimming, imputation, undersampling, feature selection, and biomarker discovery through explainable AI (XAI) models, were meticulously executed to ensure the accuracy and reliability of the results.

**Results:**

Elevated Vanillylmandelic acid (VMA) levels are markedly associated with stage 1 hypertension compared to controls. Glycerophosphorylcholine (GPC), N-Stearoylsphingosine (d18:1/18:0)*, and glycine are critical metabolites for accurate hypertension prediction. The light gradient boosting model yielded superior results, underscoring the potential of our research in enhancing hypertension diagnosis and treatment. The model’s classification metrics: accuracy (78.13%), precision (78.13%), recall (78.13%), F1-score (78.13%), and AUROC (83.88%) affirm its efficacy. SHapley Additive exPlanations (SHAP) further elucidate the metabolite markers, providing a deeper understanding of the disease’s pathology.

**Conclusion:**

This study identified novel metabolite biomarkers for precise hypertension diagnosis using XAI, enhancing early detection and intervention in the Qatari population.

## Introduction

Hypertension, a condition characterized by elevated blood pressure, is a global health concern with a rising incidence rate. In Qatar, 33% of adults are affected by this condition, which significantly increases the risk of cardiovascular diseases, stroke, renal diseases, and vision impairments (Oparil, 2018; Ventura & Lavie, 2016). The asymptomatic nature of hypertension often leads to delayed diagnosis, exacerbating its impact on public health (Gauer, 2017). Recent studies have highlighted the role of metabolic dysfunction in the pathophysiology of hypertension (Hall et al., 2024; Baker & Rutter, 2023; Vona et al., 2019; Shah et al., 2012). Altered lipid profiles, increased amino acid levels, and dysregulated glucose metabolites are among the key biomarkers associated with hypertension (Onuh & Qiu, 2021; Tanaka & Itoh, 2019). However, the specific metabolite profiles related to hypertension in the Qatari population remain underexplored, presenting a critical gap in current research.

The pathophysiological mechanisms of hypertension are multifactorial and can be delineated by examining crucial biological functions in the body that regulate blood pressure (Arnett & Claas, 2018). The metabolomics data is studied in various dimensions, methods, and protocols. Predictive models in clinical research are emerging and gaining wide attention due to higher precision and biomarker-based non-invasive diagnosis methods (Eloranta & Boman, 2022). Statistical and machine learning models deliver greater insights into understanding the relationship between metabolites and disease conditions. Despite the data modalities, machine learning algorithms tend to perform better and are advantageous in metabolomics studies (Mendez et al., 2019). In most cases, hypertension is associated with comorbidities, so identifying the right clinical factors is crucial to control the severity.

In a targeted urinary metabolomics study, the pregnancy-specific candidate metabolites are identified as the diagnostic markers for hypertension using machine learning (Varghese, 2023). The gestational age prediction model is developed with urinary metabolomics analysis to understand normal and complicated pregnancies with pre-existing hypertensive disorders. This study elucidates the advantage of proposing a non-invasive, accurate biomarker identification method (Yamauchi, 2021). Another targeted metabolomics study uses machine learning models to identify distinct patterns among the metabolites involved in endocrine forms of hypertension (EHT) and primary hypertension (PHT). However, there is a higher risk of misinterpretation when determining metabolite markers as diagnostic tools for specific diseases. This is because a single metabolite can be involved in the pathogenesis and biological functions of multiple diseases, particularly hypertension. The study utilized both classical univariate and multivariate analyses, alongside machine learning models, to delineate discriminative metabolic patterns for EHT and PHT (Erlic, 2021).

A data-driven study is conducted using multi-omics datasets for the classification of hypertension subtypes with machine learning algorithms. It addresses an important objective, distinguishing primary and secondary hypertension for precise diagnosis. Both plasma and urine samples are collected and analyzed for each subtype using 8 ML classifiers, where the best results are obtained with the random forest algorithm (Reel et al., 2022). Alongside this, the metabolite ratios are also considered an important feature in categorizing hypertension subgroups (Reel, 2022). The metabolite profiling of hypertension is crucial to characterize its impact on comorbidities such as diabetes (Leiherer, 2024), obesity (Dias-Audibert, 2020), hyperlipidemia (Fu, 2023), cardiovascular disease (Drouard, 2024), and pulmonary arterial hypertension (Alotaibi, 2023).

This study aims to address the gap in understanding hypertension by employing explainable artificial intelligence (XAI) to interpret novel metabolite biosignatures associated with hypertension in Qatar. XAI ensures accurate and transparent results while enhancing the credibility of the findings by providing clear insights into the model’s decision-making process. By leveraging advanced techniques such as liquid chromatography-mass spectrometry and comprehensive data processing methods, including imputation and feature selection, we hypothesize that analyzing the metabolomics data of hypertension profiles will identify key biomarker metabolites contributing to the development and progression of hypertension in the Qatari population. This research seeks to provide new insights and promising treatment strategies, ultimately improving the quality of life for individuals affected by hypertension.

## Materials and methods

### Qatari hypertension cohort (QBB)

**Fig. 1:**
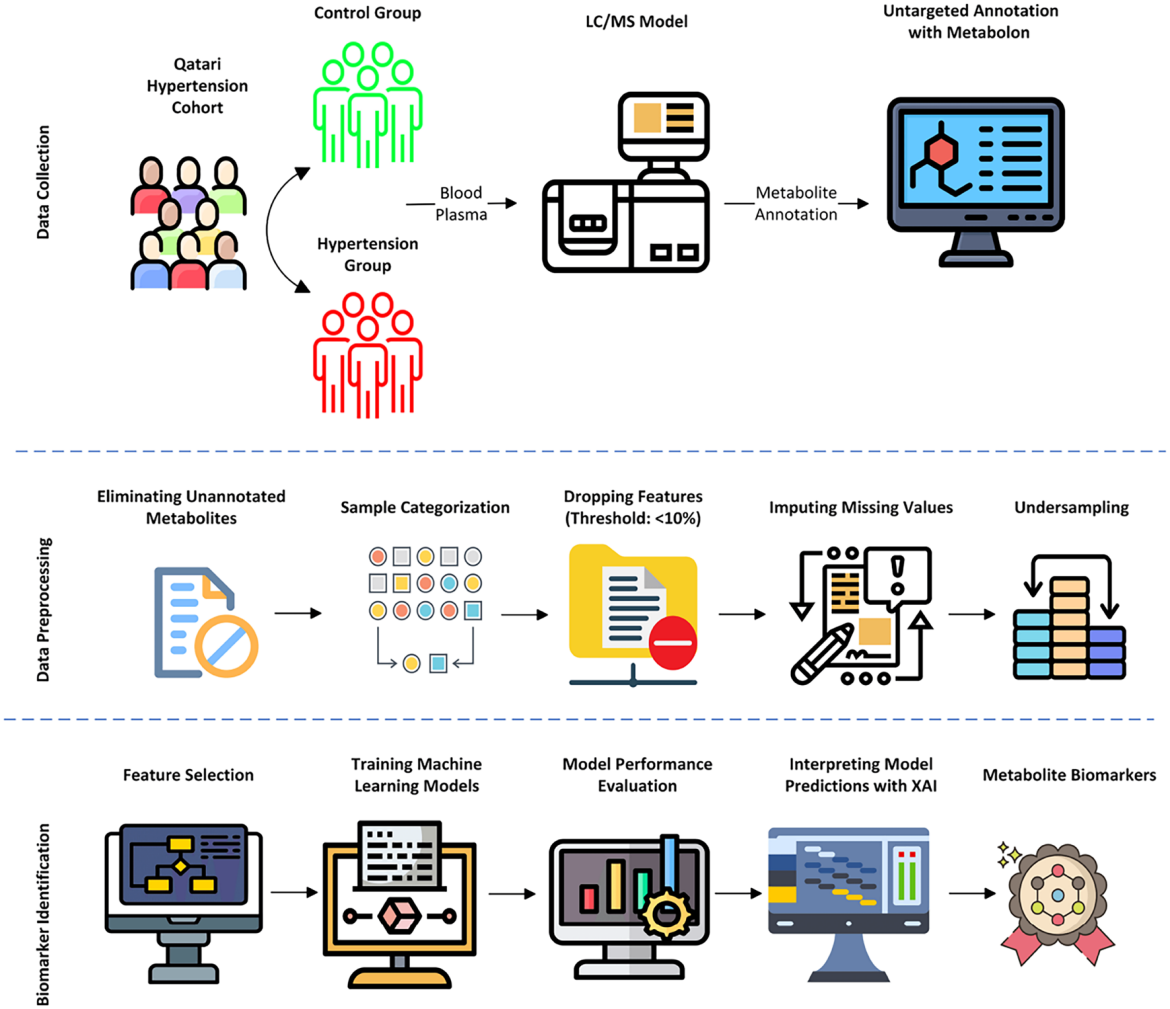
Schematic workflow of the proposed system

The metabolomics data was provided by the Qatar Precision Health Institute - Qatar Biobank (QPHI-QBB) (Al Thani et al., 2019). This study dataset contains a total sample size of 778 samples with 554 controls and 224 stage 1 hypertension cases. The demographic information shows the male in the control group (296 with a mean age of 37.80) and hypertension (133 with a mean age of 46.20), and the female in the control group (258 with a mean age of 39.22) and stage 1 hypertension (91 with a mean age of 49.29). The systematic analysis of the proposed method is depicted in Fig. 1.

### Data analysis and statistical methods

The metabolomics dataset inherently contains unannotated metabolites, missing values, and an imbalanced sample size. Statistical methods were used to process the data for further investigation. In total, 1159 metabolites were annotated by the Metabolon platform, where 222 metabolites were unnamed and removed. The number of samples was 778, with 554 control and 224 stage 1 hypertension samples in each group. The 224 samples of stage 1 hypertension were actually grouped into three subtypes (diastolic $\ge$ 80 and systolic < 130 mm Hg − 17 samples), (diastolic < 80 and systolic $\ge$ 130  mm Hg − 39 samples) and (diastolic $\ge$ 80 and systolic $\ge$ 130 mm Hg − 168 samples). According to the American Heart Association (AHA) guidelines, the above three criteria are identified as stage 1 hypertension (Flack & Adekola, 2020).

A general issue with metabolomics data is the presence of missing values. It is suggested to remove the sample or a feature if the amount of missing information is at a minimum of < 10%. Still, the missing values are present in the dataset and are handled with the imputation technique. Imputomics, a shiny web server powered by R software, is used to perform missing value imputation (Chilimoniuk et al., 2024). Metabimpute—BPCA (Bayesian Principal Component Analysis) is an efficient method for imputing missing values specific to metabolomics data. Before the feature selection step, undersampling is applied to avoid class imbalance issues during ML model preparation. The random undersampling method is applied to select 224 out of 554 control samples balancing with 224 hypertension samples.

The optimal feature subset with the best performance is identified on the cleaned dataset using HSIC Lasso (Climente-González et al., 2019). This technique delivers a combined benefit of lasso regression and kernel-based dependency scoring. It ensures retaining the metabolite markers exhibiting a non-linear relationship with the target group. HSIC maps the data into a high-dimensional space using kernel transformation methods so complex relationships can be easily identified. On the other hand, the Least Absolute Shrinkage and Selection Operator (Lasso) penalizes the less important features by shrinking its feature coefficient value to zero, ensuring sparsity and interpretability.

Six supervised machine-learning classification algorithms were trained with the final dataset containing the optimal metabolite feature subset. Logistic regression, k-nearest neighbor, support vector machine, naive bayes, light gradient boosting model, and random forest classifiers are trained with stratified k-fold cross validation (k = 5) method. Accuracy, precision, recall, and F1-score metrics calculated the performance scores of the trained classifiers. SHAP algorithm generated explanations for the model predictions out of the best performed ML algorithm trained with the metabolite dataset. It works based on cooperative game theory and describes the contribution of each feature influencing the predictions. The importance score is assigned for the features using Shapley values based on their contribution. Model agnostic explanations, fair attribution, and global and local interpretations made SHAP a powerful model-agnostic method, offers explainability to understand the predictions of black-box models (Lundberg et al., 2017).

The computational pipeline is implemented with the recent versions of scientific Python libraries for building statistical and machine-learning models. Data operations (pandas, numpy, scipy, imblearn), Feature selection (pyhsiclasso), ML model construction (sci-kit-learn), interpretation (shap), visualization (matplotlib) modules. MetaboAnalyst web server (version 6.0) is accessed to carry out functional annotation and pathway analysis (Pang et al., 2024). The protein-chemical interaction network is generated with the STITCH webserver (Szklarczyk, 2016).

## Results

In the initial metabolite dataset containing 1159 metabolites and 778 samples, the data were reduced to 570 metabolites and 448 samples, respectively, after eliminating unannotated features, thresholding missing value representations, imputation, and undersampling. The class groups—control and stage 1 hypertension are equally distributed, with 224 samples each. HSIC Lasso feature selection method identified 66 candidate metabolite markers as significant in predicting the control and hypertension samples. Each metabolite is ranked based on its importance score, where vanillylmandelate (a.k.a.) vanillylmandelic acid stands on top (1.00), followed by N-stearoyl-sphingosine(d18:1/18:0)* (0.68), 1-stearoyl-2-docosahexaenoyl-GPC(18:0/22:6) (0.52), metabolonic lactone sulfate (0.49) and pantothenate (0.448). The top 10 metabolites and their corresponding score are provided in Table 1.

**Table 1:** HSIC Lasso ranked metabolites (Top 10)

Feature	Score
vanillylmandelate (VMA)	1.000
N-stearoyl-sphingosine (d18:1/18:0)*	0.688
1-Stearoyl-2-docosahexaenoyl GPC (18:0/22:6)	0.525
Metaboloniclactonesulfate	0.497
Pantothenate	0.448
1-Stearoyl-2-arachidonoyl GPI (18:0/20:4)	0.421
2-O-methylascorbic acid	0.414
Serine	0.399
N1-Methyl-2-pyridone-5-carboxamide	0.374
Mannonate*	0.358
Hypoxanthine	0.358

**Fig. 2:**
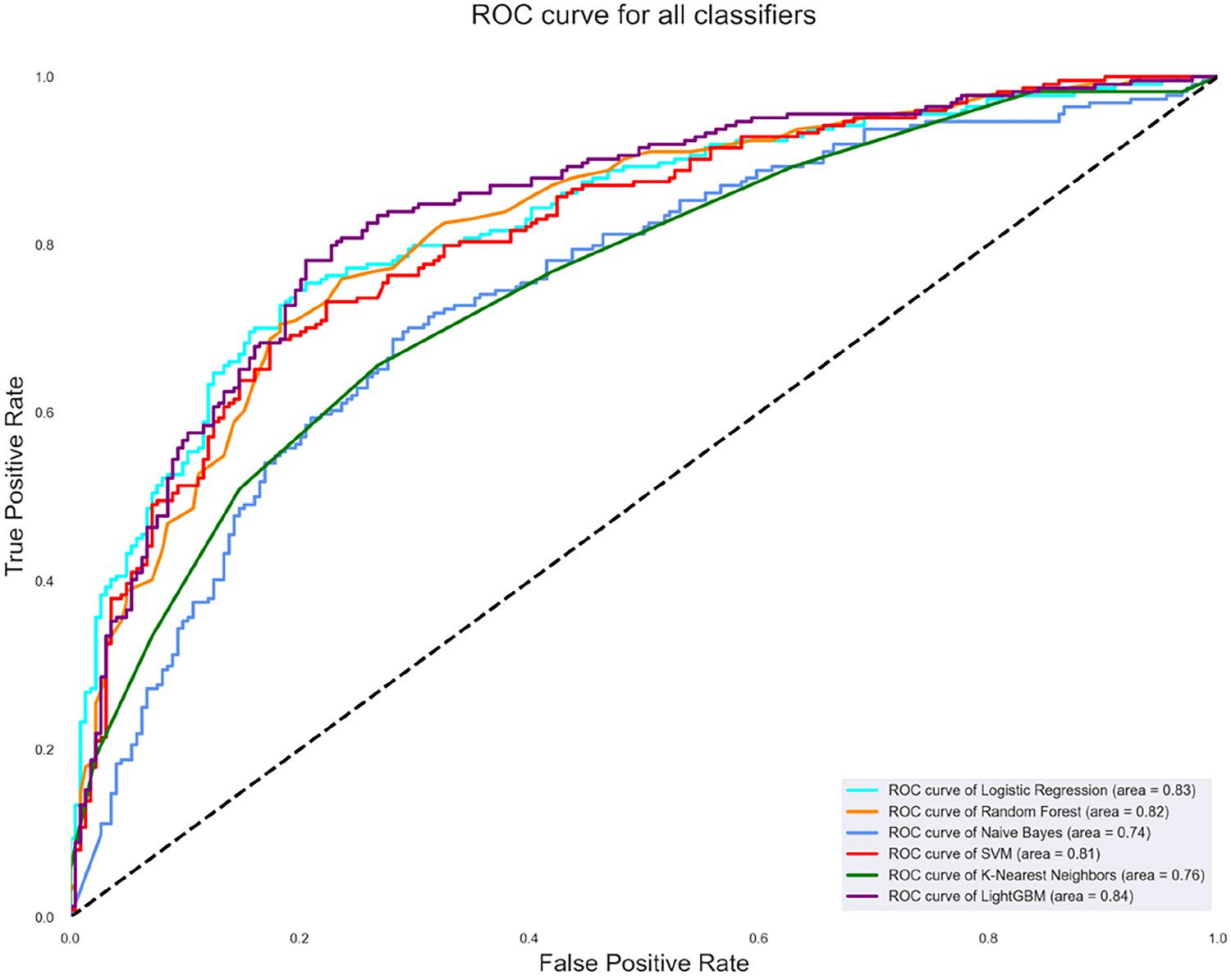
AUROC curve of the classification algorithms

The machine learning algorithms were trained with the 66 candidate metabolites and 448 samples using the stratified k-fold cross-validation (k = 5) method. The grid search technique identified the best parameter settings for each algorithm. The highest accuracy is attained by the light gradient boosting model (78.13%), followed by logistic regression (77.01%), random forest (75.45%), SVM (74.78%), naive Bayes (68.30%) and k-nearest neighbors (69.42%). Table 2 represents the algorithms’ scores for precision, recall, F1-score, and AUROC. Figure 2 depicts the combined AUROC curve of the classifiers with the best score of 83.87% by the LGBM classifier. The best model parameters of each algorithm are determined using the grid search method and are listed in Table 3.

**Table 2:** Classifier scores on the QBB metabolomics dataset

Classifier	Accuracy (%)	Precision (%)	Recall (%)	F1-score (%)	AU-ROC (%)
LR	77.01	78.40	74.55	76.43	82.85
RF	75.45	75.68	75.00	75.34	83.20
NB	68.30	74.40	55.80	63.78	74.41
SVM	74.78	75.34	73.66	74.49	81.50
k-NN	69.42	71.01	65.63	68.21	75.51
LGBM	78.13	78.13	78.13	78.13	83.88

**Table 3:** Best model parameters identified by grid search method

Classifier	Best params
Logistic Regression	{’clf__C’: 0.01}
Random Forest	{’clf__max_depth’: 30, ’clf__n_estimators’: 100}
Naïve Bayes	{ }
SVM	{’clf__C’: 1, ’clf__kernel’: ’rbf’}
K-Nearest Neighbors	{’clf__n_neighbors’: 9}
LightGBM	{’clf__learning_rate’: 0.1, ’clf__n_estimators’: }

The global and local interpretation of LGBM predictions is generated with the SHAP Explainer algorithm. The violin plot, dot plot, and bar plot illustrate the global interpretation for all samples, whereas the waterfall plot and force plot provide local interpretation of individual randomly selected sample.

**Fig. 3:**
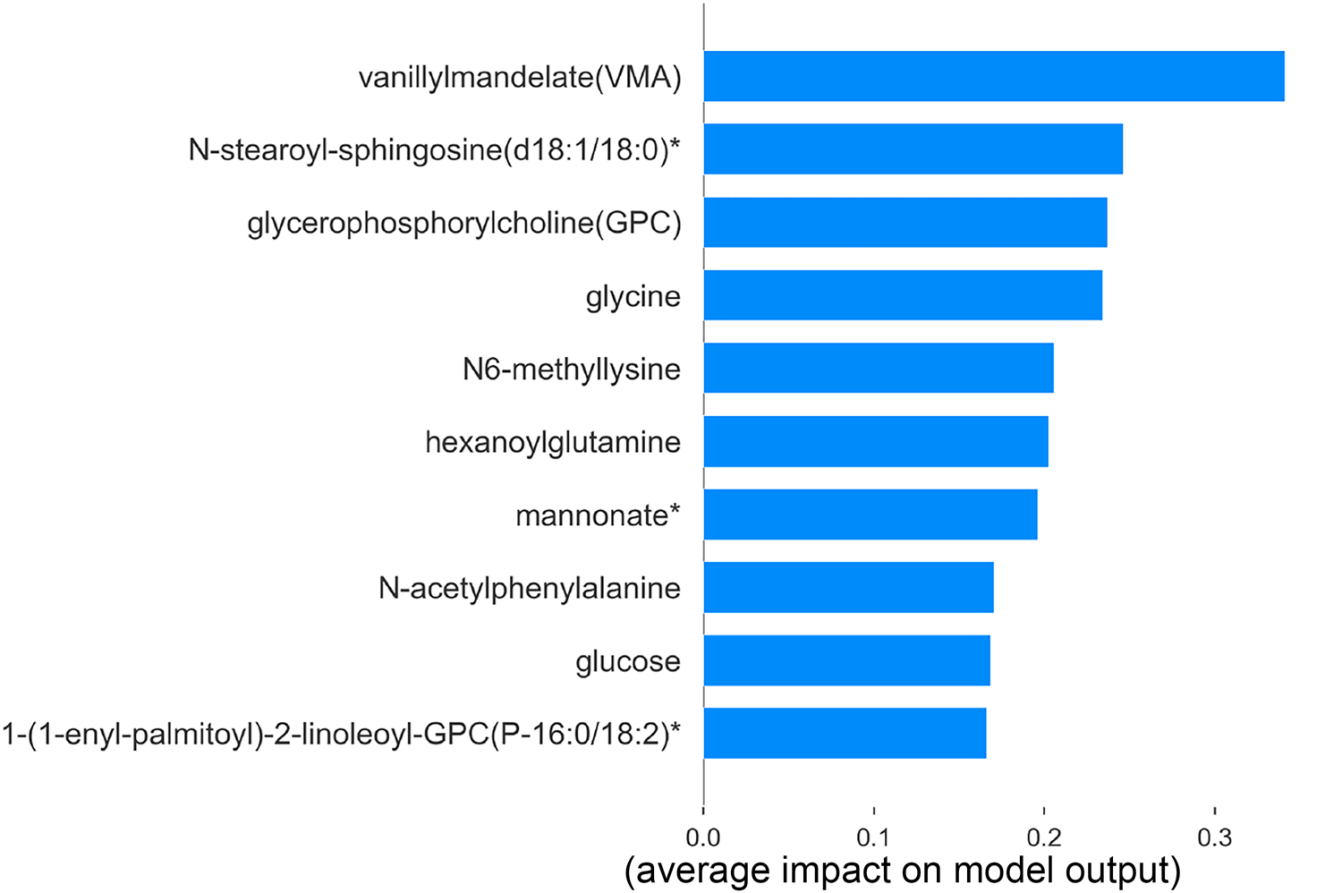
SHAP Feature importance plot

The bar plot in Fig. 3 visualizes the feature importance of the top 10 metabolites. Vanillylmandelate, N-stearoyl-sphingosine(d18:1/18:0)*, glycerophosphorylcholine, glycine, N6-methyllysine, hexanoylglutamine, mannonate*, N-acetylphenylalanine, glucose and 1-(1-enyl-palmitoyl)-2-lineleoyl-GPC(P-16:0/18:2)* is reportedly ranked as the metabolic markers of hypertension. However, the positive and negative contribution of each metabolite in classifying the hypertension and control samples has been identified with violin and dot plots.

**Fig. 4:**
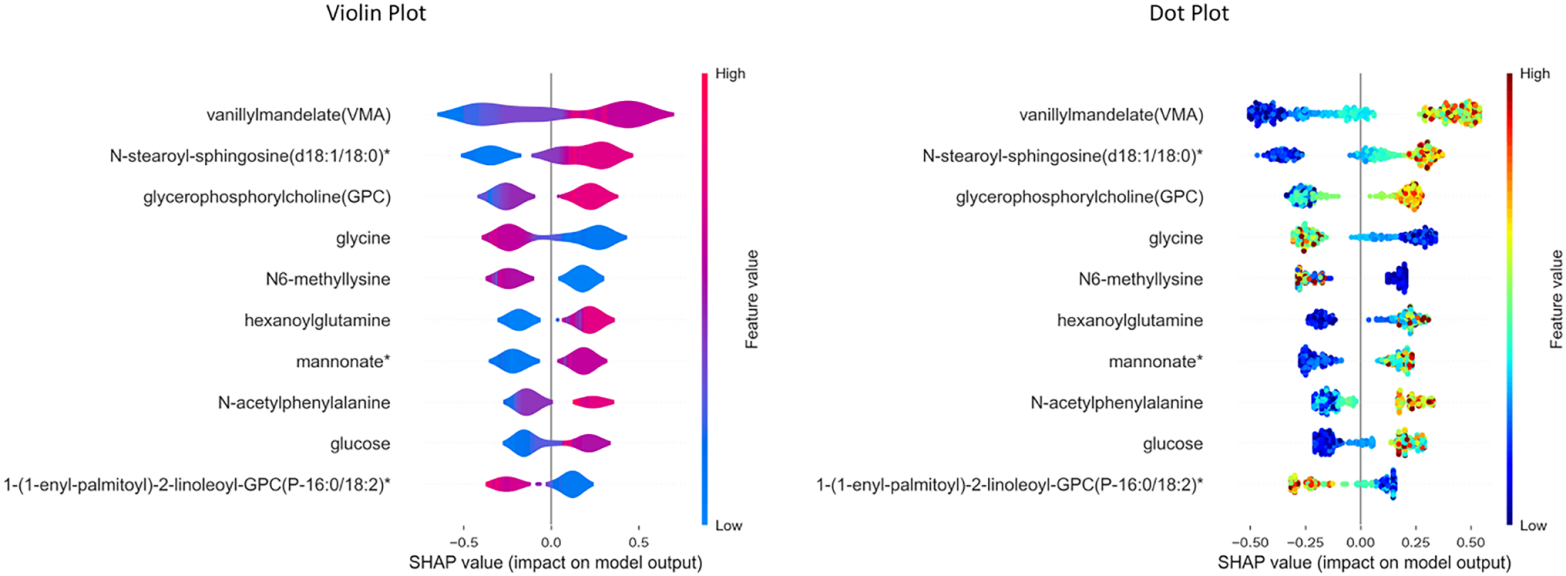
Violin and Dot plot representing global interpretation using SHAP

The model hypothesis delineates the metabolites—vanillylmandelate, N-stearoyl-sphingosine(d18:1/18:0)*, glycerophosphorylcholine, hexanoylglutamine, mannonate*, N-acetylphenylalanine, glucose influences the sample prediction as hypertension with positive SHAP value. Higher metabolite value (red) influences the prediction with a positive impact, whereas glycine, N6-methyllysine, and 1-(1-enyl-palmitoyl)-2-lineleoyl-GPC (P-16:0/18:2)* influences negative prediction as control with negative SHAP value. The decreased values (blue) of these three metabolites influence the prediction to be negative. Conversely, the reverse of the above pattern reflects on the corresponding predictions, and this can be briefly observed during local interpretation. Despite the similarity in the violin and dot plot results, represented in Fig. 4, the color gradient in the dot plot accurately showcases the contribution with colormaps.

**Fig. 5:**
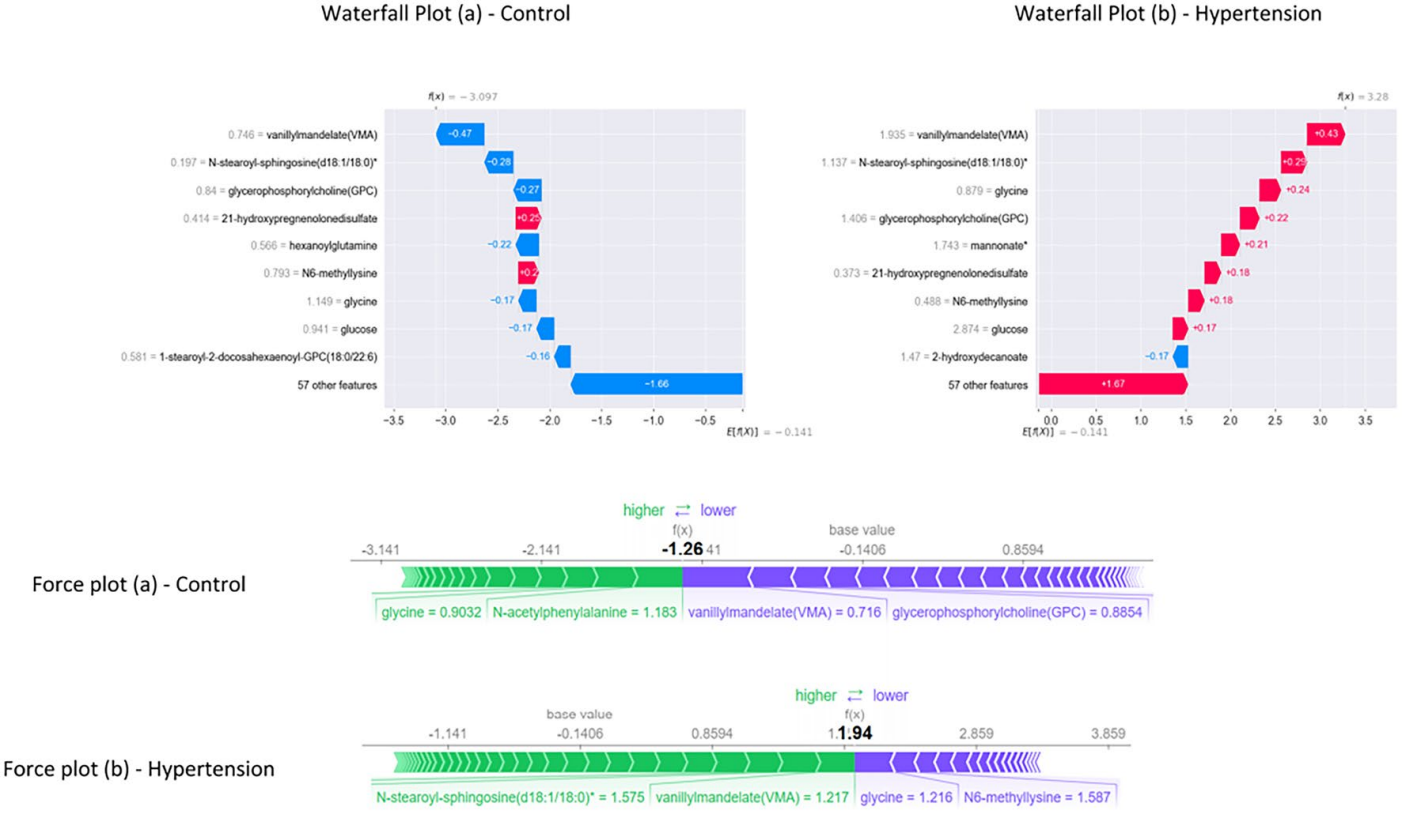
Waterfall and Force plot representing local interpretation using SHAP

The waterfall plot in Fig. 5 provides the interpretations, where the influence of metabolites on predicting the control and hypertension is transparent. In Fig. 5 waterfall plot (b), a random hypertension sample is explained, showing that higher values of the metabolites vanillylmandelate (1.935), N-stearoyl-sphingosine(d18:1/18:0)* (1.137), glycerophosphorylcholine (1.406), mannonate* (1.743), and glucose (2.874) contributing for the prediction. Notably, the decreased value of glycine (0.879) supports the prediction. Further, the control sample explanation in Fig. 5 waterfall plot (a) elucidates the decreased value of the metabolites vanillylmandelate (0.746), N-stearoyl-sphingosine(d18:1/18:0)* (0.197), glycerophosphorylcholine (0.84) and glucose (0.941) is influencing the prediction. The increased value of glycine (1.149) is another factor contributing to the prediction of the control sample. *E*[*f*(*x*)] represents the expected value of the SHAP predictions across the whole dataset and *f*(*x*) denote model prediction for the specific instance based on contributions of every feature in the dataset. The positive and negative Shapley values indicate the contribution level of each metabolite for the specific sample. The validation of the results is carried out with force plot explanations. Two random samples of hypertension and control are selected for interpretation, and the result is visualized in Fig. 5. It depicts the influence of vanillylmandelate (0.716), glycerophosphorylcholine (0.8854), and glycine (0.9032) in predicting the control (Figure 5 force plot (a)). Besides, vanillylmandelate (1.217), N-stearoyl-sphingosine(d18:1/18:0)* (1.575), and glycine (1.216) contribute to the sample prediction as hypertension [Fig. 5 force plot (b)].

**Fig. 6:**
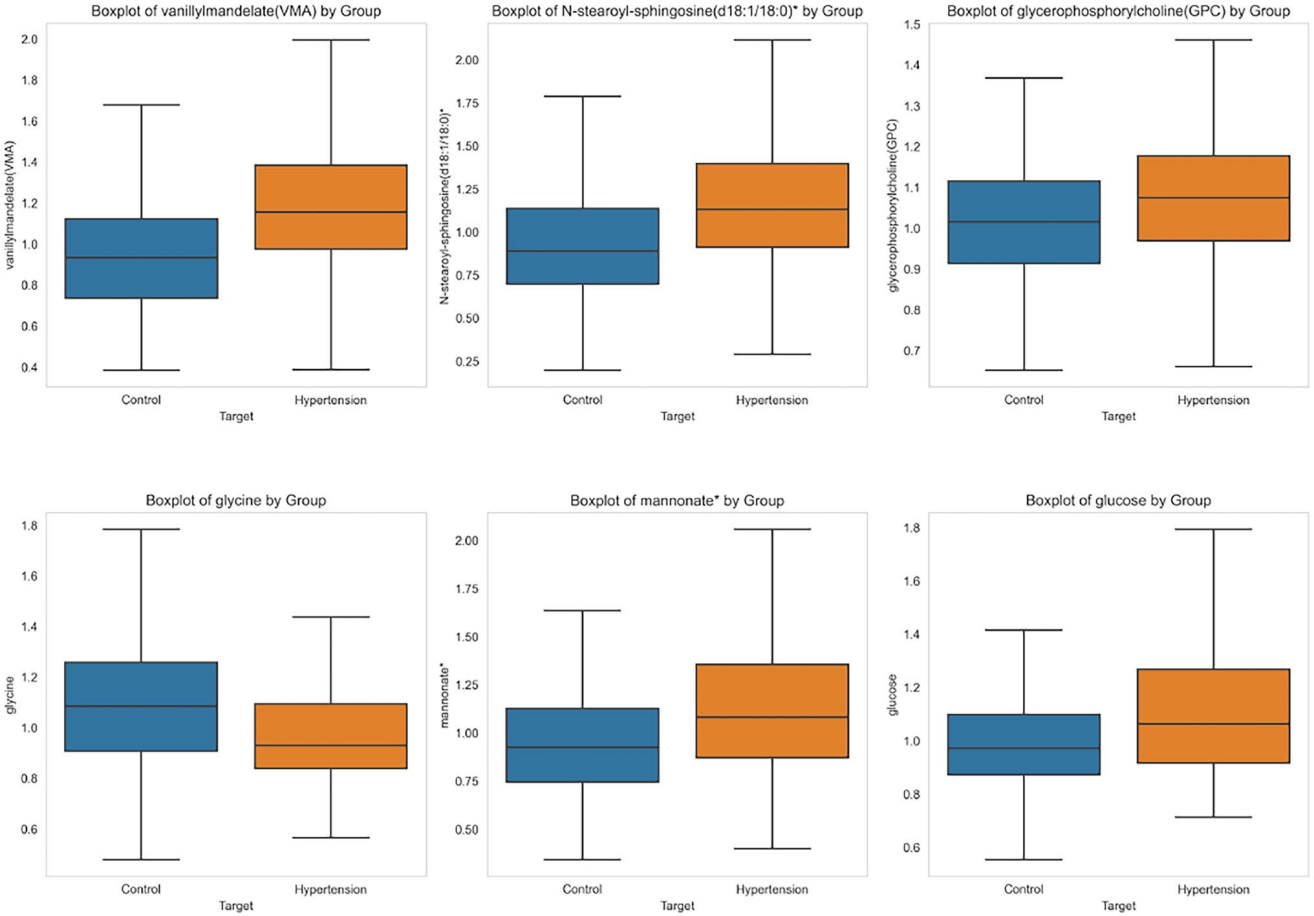
Boxplot comparison of metabolites among control and hypertension subgroups

Statistical analysis reinforces the robustness of the findings and enhances pattern visibility. The boxplot of the top 6 contributing metabolites is visualized in Fig. 6. The t-statistics and p-value of the metabolites are vanillylmandelate(VMA): t-statistic = $-$7.09, p-value = 0.00000000, N-stearoyl-sphingosine(d18:1/18:0)*: t-statistic = $-$6.72, p-value = 0.00000000 and glycerophosphorylcholine (GPC): t-statistic = $-$3.38, p-value = 0.00079321, glycine: t-statistic = 5.16, p-value = 0.00000037 mannonate*: t-statistic = $-$5.08, p-value = 0.00000055, glucose: t-statistic = $-$4.97, p-value = 0.00000094. The hypertension group observes an increase in the metabolite value for all except glycine. The mean and standard deviation of the metabolite biomarkers are tabulated below in Table 4. The statistical result validates the significance of these metabolites in the prediction of hypertension.

**Table 4:** Mean and standard deviation of the metabolite biomarkers

Group	Mean	S.D	Group	Mean	S.D
Vanillylmandelate			Glycine		
Control	0.97	0.31	Control	1.13	0.32
Hypertension	1.198	0.36	Hypertension	0.99	0.26

N-stearoyl-sphingosine (d18:1/18:0)*			Mannonate*		
Control	0.95	0.36	Control	0.99	0.37
Hypertension	1.20	0.43	Hypertension	1.20	0.50

Glycerophosphorylcholine			Glucose		
Control	1.01	0.15	Control	1.01	0.25
Hypertension	1.07	0.19	Hypertension	1.21	0.52

### Metabolite functional annotation and chemical-protein interactions

**Fig. 7:**
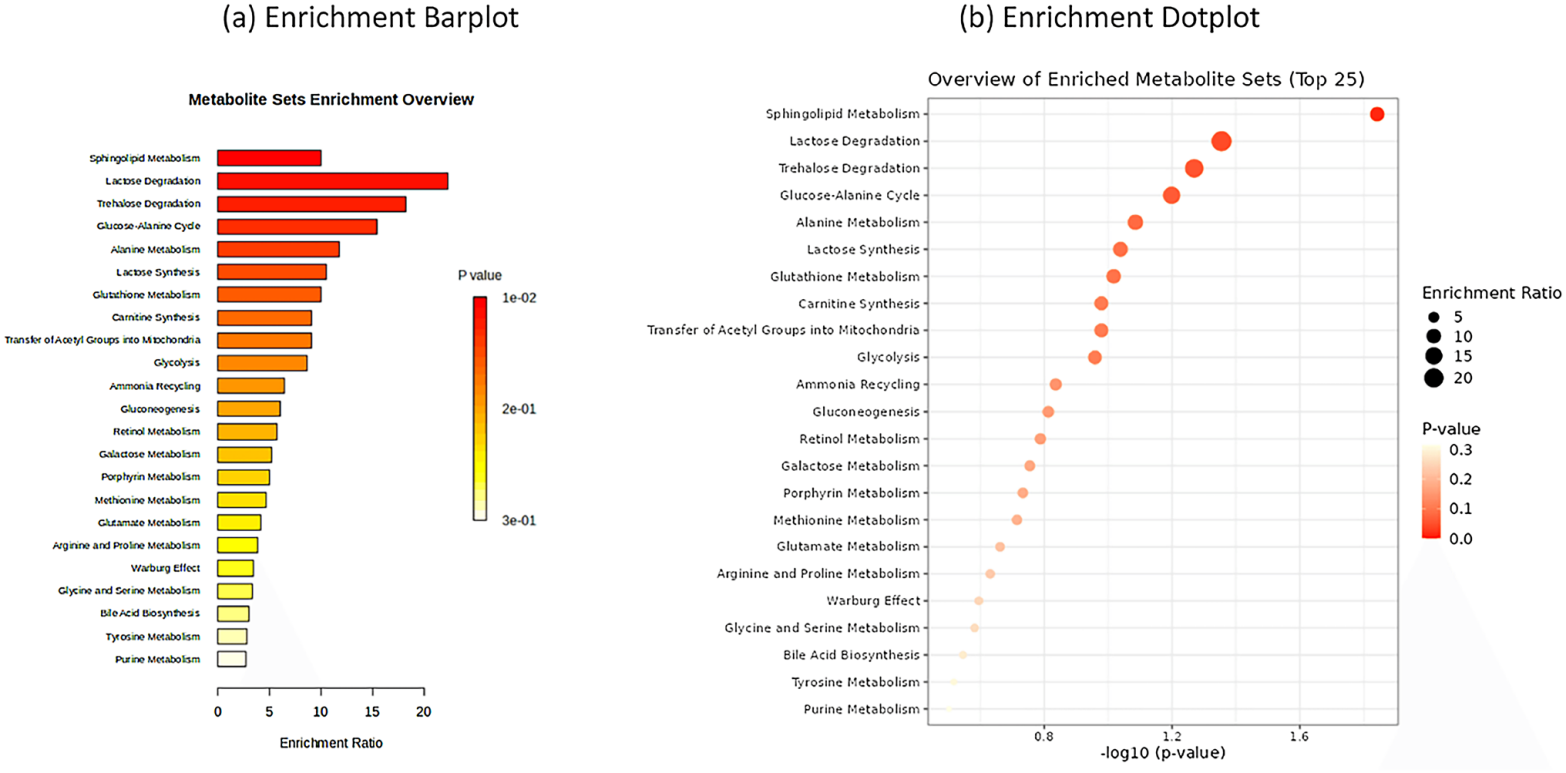
Enrichment plots of top metabolite markers

The enrichment analysis of the top 10 metabolites ranked by SHAP during feature importance analysis is performed using the MetaboAnalyst web server. The HMDB IDs of the metabolites are the inputs, and the over-representation analysis is conducted on The Small Molecule Pathway Database (TSMPD). The enriched metabolites, sorted on p-value, are visualized as bar plots and dot plots in Fig. 7a and b, respectively.

**Table 5:** Table of Metabolites, Super Pathways, and Sub Pathways

Metabolites	Super pathway	Sub pathway
Vanillylmandelate (VMA)	Amino Acid	Tyrosine Metabolism
N-stearoyl-sphingosine (d18:1/18:0)*	Lipid	Ceramides
Glycerophosphorylcholine (GPC)	Lipid	Phospholipid Metabolism
Mannonate*	Xenobiotics	Food Component/Plant
Glycine	Amino Acid	Glycine, Serine and Threonine Metabolism
Glucose	Carbohydrate	Glycolysis, Gluconeogenesis, and Pyruvate Metabolism

**Fig. 8:**
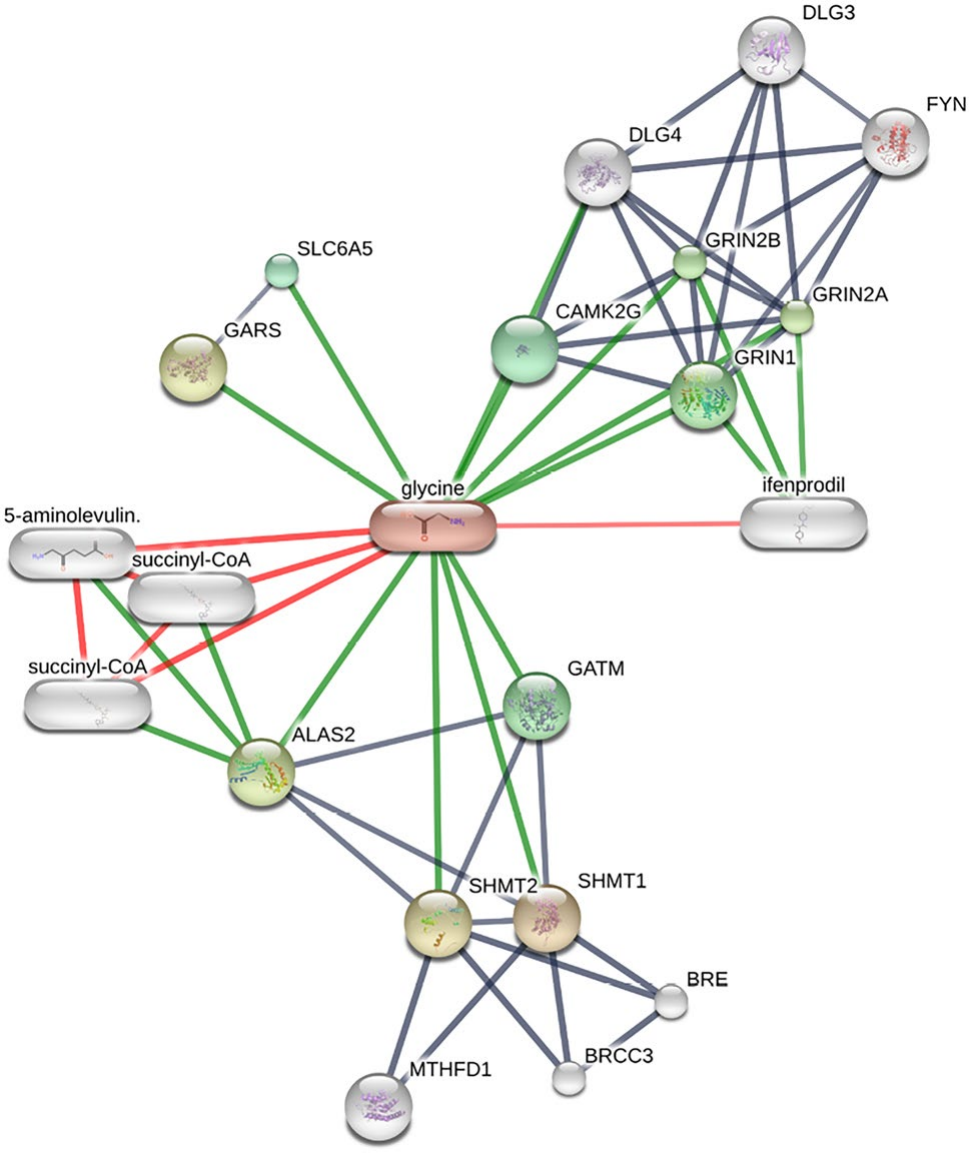
Chemical-protein interaction of top metabolites

Sphingolipid metabolism has the highest significance among other enriched metabolites. The role of sphingolipids is vital in cell membrane structure and cellular signaling. It involves physiological and pathological activities in hypertension and cardiovascular disease. Vascular tone regulation is an important mechanism where S1P binds to its receptors (S1PR1-5), affecting the endothelial cells and smooth muscle cells in blood vessels. Studying the complex, intricate S1P signaling pathways holds promising solutions for treating hypertension. The super and sub pathways of the metabolite biomarkers are represented in Table 5. The chemical-protein interaction for known and predicted entities is represented as a network plot in Fig. 8 using the STITCH web server. The highest number of protein interactions is observed in the glycine.

## Discussion

This study leverages the capability of explainable artificial intelligence to identify potential novel metabolite biomarkers associated with hypertension in the Qatari population. By employing advanced metabolomics techniques and a rigorous data processing pipeline, our findings provide important insights into the pathophysiology of hypertension, offering potential avenues for early diagnosis and intervention.

The analysis identified several metabolites with significant associations with hypertension. Spiked levels of Vanillylmandelic acid, N-stearoyl-sphingosine(d18:1/18:0)*, and Glycerophosphorylcholine were prominently linked to hypertensive cases, while Glycine showed an inverse relationship. These metabolites play vital roles in various biochemical pathways, underscoring their potential as biomarkers for hypertension.

Machine learning algorithms were crucial in identifying metabolite markers of hypertension, with VMA emerging as the top biomarker using the HSIC Lasso feature selection algorithm. A total of 66 significant features were then analyzed, where LGBM outperformed other models in every metric, with a higher accuracy of 78.12% (Table 2). Accuracy is an important evaluation method in class-balanced datasets. The AU-ROC curve in Figure 2 shows the lucid performance of LGBM with 83.88%. The SHAP model is employed to delineate the underlying predictions and influence of metabolites on the best-performed LGBM.

Abnormal VMA levels can be associated with conditions that involve dysregulated catecholamine metabolism, such as pheochromocytoma, potentially leading to hypertension (Yin et al., 2021), providing a potential diagnostic biomarker for hypertension. N-Stearoylsphingosine and Glycerophosphorylcholine are involved in lipid metabolism, bridging the role of lipid signaling in hypertension. Glycine, typically associated with reduced cardiovascular risk, further highlights the metabolic complexities underlying the disease (Rebholz et al., 2018).

VMA is identified as a key diagnostic biomarker for certain conditions involving excessive catecholamine secretion, such as pheochromocytoma, causing severe secondary hypertension. The increased levels of VMA in these cases highlight the direct link between catecholamine metabolism and the pathophysiology of hypertension, particularly in secondary forms where overproduction of catecholamines leads to persistent high blood pressure. This suggests that VMA could be a useful biomarker in distinguishing between different etiologies of hypertension, However, limited experimental evidence exists regarding the direct pathological role of VMA in hypertension, indicating the need for further research (Zhang, 2024).

Lipid metabolism plays a significant role in hypertension. N-Stearoylsphingosine, a sphingolipid metabolite, is crucial for maintaining cell membrane integrity and regulating vascular tone through its involvement in the cellular signaling pathway. The spike of this metabolite in hypertensive patients supports the notion that lipid signaling is integral to vascular health and aligns with research on lipid metabolism in cardiovascular diseases. Furthermore, the connection between tyrosine metabolism, a super pathway of VMA, and blood pressure regulation through catecholamines and thyroid hormones adds complexity to the metabolic basis of hypertension (Deng et al., 2021). This multifactorial perspective highlights how disturbances in metabolic pathways, including both catecholamine and lipid metabolism, contribute to hypertension.

The role of other metabolites, such as glycine, in hypertension adds another layer of complexity to the disease. Glycine, typically associated with reduced cardiovascular risk, has been observed to interact with other metabolic pathways that influence blood pressure regulation. The intricate balance of these metabolic processes underscores the multifactorial nature of hypertension, where dysregulation in one pathway can have significant downstream effects on blood pressure control.

The relationship between metabolites and hypertension underscores the disease’s complexity. Elevated glycerophosphorylcholine levels, involved in phospholipid metabolism, are linked to hypertension, suggesting alterations in cell membrane composition and signaling pathways (Zhang, 2024). Conversely, glycine, typically associated with reduced cardiovascular risk, shows protective effects against hypertension, with lower levels observed in hypertensive individuals indicating its potential as a therapeutic target (Imenshahidi & Hossenzadeh, 2022). Additionally, VMA is involved in sphingolipid metabolism, both correlate with hypertension, unraveling the multifactorial nature of the disease where disruptions in catecholamine and lipid metabolism play significant roles in blood pressure regulation.

The interaction of various metabolites with their super and sub-pathways highlights the complex nature of hypertension. VMA derived from tyrosine metabolism, reflects dysregulation in catecholamine pathways and its association with hypertension. N-Stearoyl-sphingosine (d18:1/18:0)*, a component of ceramide synthesis, underscores the role of lipid signaling in vascular health, while glycerophosphorylcholine in phospholipid metabolism emphasizes the importance of membrane integrity and cellular signaling. Glycine, involved in glycine, serine, and threonine metabolism, is linked to reduced cardiovascular risk, supporting its potential as a therapeutic target. Mannonate, a food-derived xenobiotic, and glucose from glycolysis and gluconeogenesis, further illustrate the diverse metabolic influences on hypertension.

Enrichment analysis of top biomarkers displayed by SHAP global interpretation revealed the interaction of sphingolipid metabolism. It has the highest significance among other enriched metabolites, is involved in sphingolipids is vital in cell membrane structure and cellular signaling. Despite the lesser statistical significance, these findings stand as current evidence for future investigations. The highest number of chemical-protein interactions is observed in the glycine metabolite, illustrated in Fig. 8.

Our integrated AI-driven approach to metabolite interactions enhances our understanding of hypertension’s complex etiology and offers potential pathways for novel diagnostic and therapeutic approaches. Collectively, these findings underscore the need for continued research into metabolic profiling as a tool for identifying and managing hypertension, paving the way for more effective precision medicine approaches.

## Limitations and future perspectives

The focus on a specific population may limit the generalizability of our findings. Future research should aim to validate these biomarkers in diverse cohorts to ensure broader applicability. While LC/MS is a powerful tool for metabolite identification, its limitations in sensitivity and specificity must be considered. Exploring complementary techniques and expanding the metabolite panel could provide a more comprehensive understanding of hypertension metabolic underpinnings.

Further research should also investigate the mechanistic roles of the identified metabolites in hypertension. Longitudinal studies assessing changes in these biomarkers over time and in response to treatment could offer valuable insights into their potential as therapeutic targets. Additionally, integrating other omics data, such as genomics and proteomics, could enhance the robustness of biomarker discovery and provide a holistic view of hypertension pathophysiology.

## Conclusion

This study provides significant advancements in hypertension research by integrating explainable artificial intelligence (XAI) to uncover novel biomarker metabolites within the Qatari population. Increased levels of Vanillylmandelic acid, N-stearoyl-sphingosine(d18:1/18:0)*, and Glycerophosphorylcholine emerged as robust indicators of hypertension, while Glycine was inversely associated. By employing advanced liquid chromatography-mass spectrometry and the HSIC Lasso feature selection method, we rigorously identified 66 key metabolites, with the light gradient boosting model (LGBM) demonstrating exceptional predictive accuracy. The use of SHapley Additive exPlanations offered transparent insights into metabolite contributions, enhancing the reliability of the model’s predictions. These findings not only provide ethnic-specific insights but also align with global cardiovascular research, underscoring the critical role of lipid metabolism in hypertension. Our research positions these metabolites as promising diagnostic and therapeutic biomarkers, paving the way for early, more effective intervention strategies that could significantly reduce hypertension-related comorbidities and improve patient outcomes.

## Data Availability

Qatar biobank data can be accessed upon request using the online portal: (https://www.qatarbiobank.org.qa/research/how-apply). This portal is subject to approval by the QBB IRB Committee. This study used the Qatar biobank data under the project (QF-QBB-RES-ACC-00095).
